# Absorption of Water Vapor by Bambus[6]uril and a Density Functional Theory Study of Its Aqua Complexes

**DOI:** 10.3390/molecules28237680

**Published:** 2023-11-21

**Authors:** Pana Turebayeva, Alexey N. Guslyakov, Svetlana A. Novikova, Andrei I. Khlebnikov, Ekaterina A. Befus, Evgeniy P. Meshcheryakov, Abdigali A. Bakibaev, Lyazat Kusepova, Nazira Kassenova, Sarzhan Sharipova, Rakhmetulla Yerkassov

**Affiliations:** 1 Department of Chemistry, L.N. Gumilyov Eurasian National University, Astana 010008, Kazakhstan; kusepova71@mail.ru (L.K.);; 2 Faculty of Chemistry, National Research Tomsk State University, 634050 Tomsk, Russia; guslyakov.aleksej@bk.ru (A.N.G.); ekaterina.befus@mail.ru (E.A.B.); meevgeni@mail.ru (E.P.M.);; 3 Kizhner Research Center, Tomsk Polytechnic University, 634050 Tomsk, Russia; san22@tpu.ru (S.A.N.);; 4 Department of Chemistry and Biotechnology, Sh. Ualikhanov Kokshetau University, Kokshetau 020000, Kazakhstan; nazira09_83@mail.ru; 5 Department of Pharmaceutical and Toxicological Chemistry, Pharmacognosy and Botany, Asfendiyarov Kazakh National Medical University, Almaty 050000, Kazakhstan

**Keywords:** bambus[6]uril, water absorption, DFT calculations, water desorption, aqua complex

## Abstract

The absorption/desorption of water vapor by bambus[6]uril (Bu[6]) has been studied. According to kinetic experiments, the absorption capacity of Bu[6] is 4 moles of water per 1 mole of Bu[6] with the absorption duration of 20 min and the complete desorption duration of 100 min. Experimental rate constants for water vapor absorption and desorption by Bu[6] have been determined to be 0.166 min^−1^ and 0.0221 min^−1^, respectively. The obtained results are in agreement with theoretical calculations using the DFT method. A hypothetical structure of bambus[6]uril tetrahydrate (Bu[6]·4H_2_O) has been proposed based on the experimental and DFT data.

## 1. Introduction

**Figure 1:**
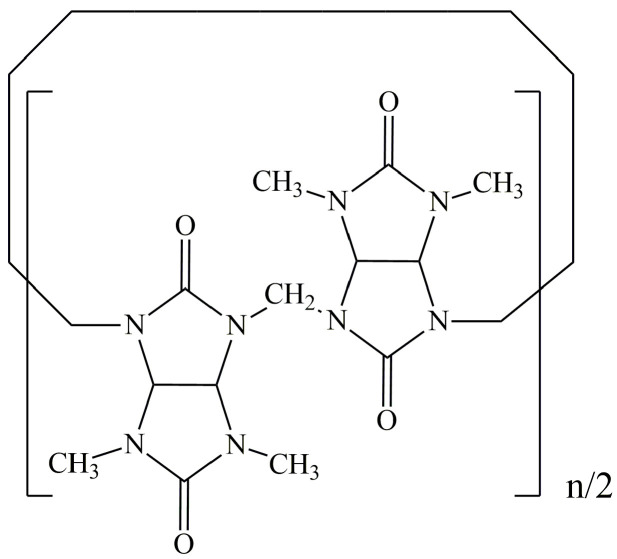
The structure of bambus[n]uril, where n = 4, 6.

Bambus[n]urils with n = 4, 6 (Bu[n]) were discovered relatively recently by a group of Czech scientists [1]. Bu[n] represents a new class of macrocyclic compounds consisting of N,N’-disubstituted glycoluril units connected through a series of methylene bridges (Figure 1).

These macrocycles combine the structural characteristics of both cucurbit[n]urils and hemi-cucurbit[n]urils [1]. With a height of 12.7 Å, Bu[6] possesses a significantly deeper cavity compared to cucurbit[n]urils, which have a height of 9.1 Å. It also exhibits a high affinity to negatively charged ions [2,3,4,5]. Current research on the chemistry of bambus[n]urils focuses on their utilization as efficient and selective absorbents for anions [6,7,8] owing to their propensity for host–guest interactions [9,10] and the formation of supramolecular complexes. In 2023, it was found that bambus[6]uril-treated porous titanium nickelide exhibits high biocompatibility and low toxicity [11].

The synthesis of bambus[6]uril occurs in a hydrochloric acid solution [2], resulting in the inclusion of residual traces of water in Bu[6]. The aqua complexes formation often causes a hydrolysis of different guest molecules within the cavity or hinders a guest release from the supramolecular complex. Thus, the presence of strongly bound water molecules in the inner cavity of Bu[6] complicates the formation of bambusuril-based inclusion compounds. While the host–guest chemistry of bambusurils continues to develop actively, the Bu[n] capacity towards such a simple and ubiquitous solvent like water remains unexplored.

Density Functional Theory (DFT) calculations are widely used for modeling supramolecular systems and the estimation of interaction energies between host and guest molecules. For example, a theoretical investigation of encapsulated systems based on Bu[6] and halide anions has been conducted recently [12].

In light of the aforementioned points, the objective of our study was to investigate the absorption/desorption capacity of bambus[6]uril towards water, determine the kinetic parameters of these processes, and explore the hydration of bambus[6]uril using the DFT method.

## 2. Results and Discussion

### 2.1. Synthesis of Bambus[6]uril

Bu[6] was synthesized following the traditional approach [2] through the cyclization reaction of 2,4-dimethylglycoluril with paraformaldehyde in 6 M HCl, thus resulting in the product yield of 25%. The obtained Bu[6] was further treated with hydroiodic acid, followed by subsequent processing with hydrogen peroxide to remove chloride ions [13].

The ^1^H NMR spectroscopy analysis of Bu[6] (DMSO-d_6_/CDCl_3_ (1:1), TMS, δ, ppm) showed the following signals: 5.29 (s, 12H) corresponding to the methine protons, 5.06 (s, 12H) assigned to the methylene protons, and 2.51 (s, 36H) corresponding to the methyl groups. In the ^13^C NMR spectrum (DMSO-d_6_/CDCl_3_ (1:1), TMS, δ, ppm) of the product, the following signals are present: 159.32 and 158.45 (the nonequivalent carbonyl fragments), 67.82 (the methine carbon atoms), 48.78 (the methylene groups), and 31.06 (the methyl carbon atoms). The IR spectrum indicated absorption bands at 3381 cm^−1^ (traces of absorbed water), 1722 cm^−1^ (ν C=O of the urea moiety), 1703 cm^−1^ (ν C=O of the 1,3-dimethylurea fragment), 1402 cm^−1^ (δ C–H of –CH_3_), 1260 cm^−1^ (δ C–H of –CH_2_–), 1129 cm^−1^ (ν C–C), and 793 cm^−1^ (ν N–C–N). These chemical shifts and IR bands are in agreement with the literature data [1,14].

### 2.2. Determination of the Dynamic Absorption Capacity of Bambus[6]uril for Water

**Figure 2:**
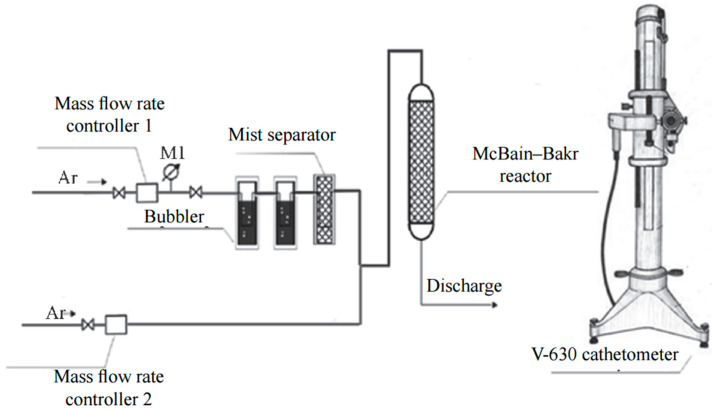
Scheme of the absorption setup for studying the kinetics of water vapor absorption/desorption.

The absorption capacity of bambus[6]uril for water was determined using a gravimetric method with McBain–Bakr quartz spring balances (Figure 2) [15]. The sensitivity of the balances was 2.9 × 10^−3^ g·mm^−1^. An optimal amount of absorbent (0.02–0.03 g) was carefully chosen to ensure a single-layer arrangement of the absorbent in the quartz cup. Prior to conducting the absorption experiments, Bu[6] was regenerated to remove any residual water by heating at 200 °C under a continuous flow of argon gas with impurity content not exceeding 10 ppm. Dehydration of Bu[6] occurs at 100–130 °C, which was confirmed by the data of the DSC analysis [14]. The argon gas was supplied at a rate of 5 L·h^−1^ for a duration of 1 h.

To initiate the absorption process of water vapor, the sample was exposed to argon gas passed through two Dreschel flasks containing distilled water (gas humidity 100%) (Figure 2). To eliminate the potential influence of the water vapor delivery rate on the external surface of Bu[6], a series of preliminary experiments were conducted using progressively increasing flow rates. Based on the experimental findings, a gas flow rate of 30 L·h^−1^ was selected as the optimal condition for the absorption process.

**Figure 3:**
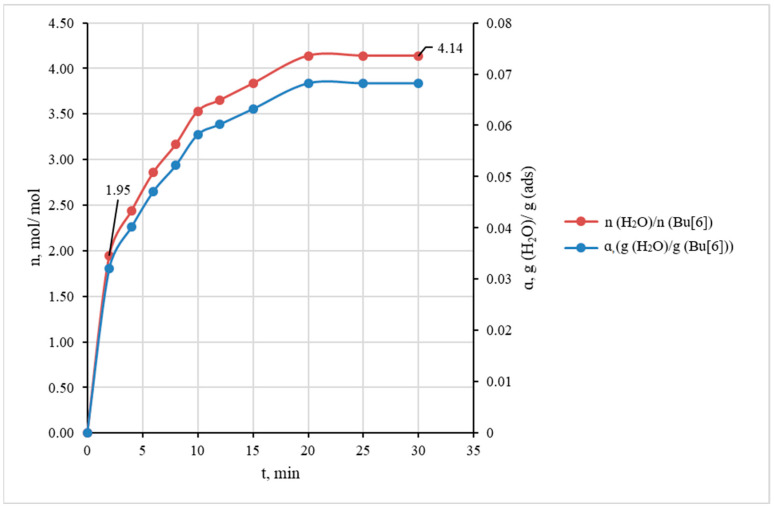
The kinetic curves of water vapor absorption expressed in terms of mass ratio g(H_2_O)/g(Bu[6]) and molar ratio n(H_2_O)/n(Bu[6]) (gas flow rate of 30 L·h^−1^).

Upon reaching the saturation with water upon the absorption by bambus[6]uril, the desorption process was initiated by introducing dry argon flow at a rate of 10 L·h^−1^. Figure 3 shows the kinetic curve of water absorption by the investigated Bu[6] sample. The curve was built based on the average data of three individual experiments at 25 °C.

From the absorption curve (Figure 3) it is evident that the highest rate of water absorption occurs within the initial 2 min period when the Bu[6] sample absorbs approximately half of its maximum capacity for H_2_O. For clarity, the kinetic curve in Figure 3 is also presented in terms of molar ratio n(H_2_O)/n(Bu[6]). These experimental data show that the maximum absorption capacity corresponds to 4 moles of water per mole of bambus[6]uril, with the saturation being attained after approximately 20 min.

To simulate the dynamic absorption of water vapor on the absorbent surface, the widely used pseudo-first-order model was applied. For isothermal absorption under a constant partial pressure, the absorption process can be described by Equation (1) [16]:

(1) $$\frac{d\alpha }{dt}=k_{abs}\left(\alpha_{max}-\alpha \right),$$

where α is the mass ratio g(H_2_O)/g(Bu[6]) measured at time *t*; α*_max_* is the maximum value of α achieved upon the sample saturation with water; and *k_abs_* is the absorption rate constant.

The integral form of this kinetic equation is a linear function of *t* (2):

(2) $$\ln\left(\alpha_{max}-\alpha \right)=-k_{abs}t+\ln\;\alpha_{max}.$$

From the linear plot (Figure S1) based on Equation (2), the value of the absorption rate constant *k_abs_* = 0.166 ± 0.008 min^−1^ was estimated.

**Figure 4:**
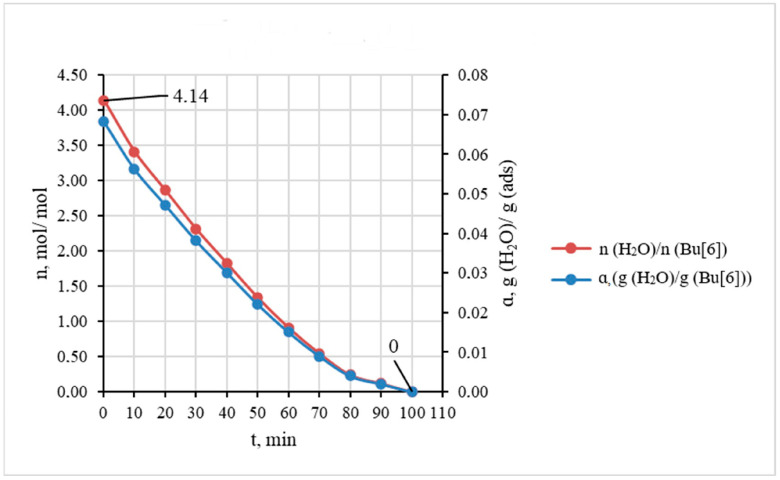
The kinetic curves of water desorption expressed in terms of mass ratio g(H_2_O)/g(Bu[6]) and molar ratio n(H_2_O)/n(Bu[6]) (gas flow rate 10 L·h^−1^).

Figure 4 illustrates the curve of water desorption from Bu[6] obtained from the average data of three individual experiments. This curve indicates that the complete removal of bound water from the sample was attained within 100 min at 25 °C and a flow rate 10 L·h^−1^ of dry argon.

The rate constant for water desorption can be determined from the pseudo-first-order model (3):

(3) $$\ln\;\alpha =-k_{des}t+\ln\;\alpha_{max},$$

where *k_des_* is the desorption rate constant.

The linearity according to Equation (3) was observed at up to 50 min of the experiment’s duration (Figure S2). The desorption rate constant *k_des_* = 0.0221 ± 0.0013 min^−1^ was calculated from this model. The errors of *k_abs_* and *k_des_* were estimated using the variances of linear approximations (Figures S1 and S2) with the confidence level of 0.95.

According to our experiments, the absorption of water vapor by Bu[6] and its desorption follow the first-order kinetic equations (the *r*^2^ values are 0.988 and 0.989 for models (2) and (3), respectively), although the aqua complex formation should be a multi-stage process (see below). These experimental results support diffusion control of the kinetics. The ratio α*_max_* observed upon saturation corresponds to the formation of relatively stable aqua complex Bu[6]·4H_2_O. Nevertheless, this mass ratio shows that Bu[6] upon hydration contains just about 7% of water, being hydrophobic in nature.

### 2.3. Investigation of Bambus[6]uril Hydration Using the DFT Method

In order to investigate the potential incorporation of water molecules into the cavity of Bu[6] through gas-phase sorption, we performed quantum chemistry calculations using the DFT method. These calculations were conducted for the Bu[6] molecule itself and for the inclusion compounds Bu[6]·nH_2_O (where n ranged from 1 to 5). To optimize the geometry of both Bu[6] and the supramolecular complexes, a modern variant of DFT, the composite method B97-3c [17], was employed. This method is low cost and quite accurate, being well suited for handling large molecular systems. Also, it accounts for dispersion interactions and corrects the basis set superposition errors (BSSE), which is especially important for DFT calculations of supramolecular systems and coordination compounds.

**Figure 5:**
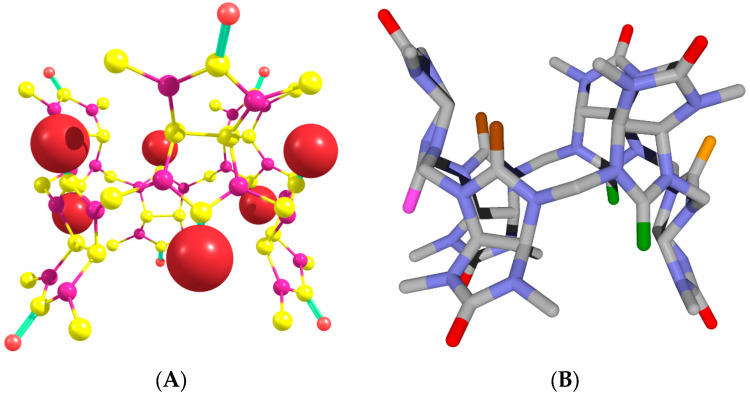
Structure of Bu[6] molecule optimized using the B97-3c method. Hydrogen atoms are omitted for clarity. (**A**) The equatorial carbonyl oxygen atoms are represented by larger red spheres, while the peripheral oxygen atoms are depicted as smaller red spheres. (**B**) The peripheral oxygen atoms are shown as red cylinders. In relation to the equatorial oxygen atom highlighted by the pink cylinder, the other equatorial oxygen atoms are positioned in *syn*-, *gauche*-, or *trans*-orientations, as indicated by the green, brown, or orange cylinders, respectively.

The geometry optimization of Bu[6] in the gas phase led to the structure shown in Figure 5. Within the Bu[6] molecule, six of the twelve carbonyl oxygen atoms are positioned at the molecule’s periphery, while the remaining six O atoms are located near the central plane of the twenty-four-membered macrocycle. Oxygen atoms of the latter type can be referred to as equatorial (Figure 5A).

To define the terminology used in this study, it is essential to establish the relative arrangement of the equatorial oxygen atoms. The *syn* position refers to two equatorial oxygen atoms located on the same side of the macrocycle as the current equatorial atom; the *gauche* position denotes the pair of equatorial oxygen atoms closest to the current one from the opposite side of the macrocycle; lastly, the *trans* position represents the carbonyl oxygen atom that is farthest from the current equatorial atom. In Figure 5B, the respective positions are visually indicated and will be designated as *s*, *g*, and *t*.

According to our DFT results, the outer diameter of the Bu[6] molecule in the gas phase calculated as twice the average distance of the three peripheral oxygen atoms from their geometric center equals 11.3 Å. The distance between the geometric centers of three “upper” and three “lower” peripheral oxygen atoms is 9.1 Å. This value can be regarded as the height of the molecule. However, it is reasonable to measure the effective Bu[6] diameter and height by adding two van der Waals radii of the oxygen atom (2 × 1.40 Å) to these values, which gives 14.1 and 11.9 Å, respectively. It would be also informative to calculate a diameter of the inner cavity within the Bu[6] molecule. We evaluated this characteristic as twice the average distance of the twelve central hydrogen atoms (proton nuclei) in CH-CH fragments of glycoluril moieties from their geometric center. Such a calculation gives the diameter of 7.0 Å. Considering that the effective size of the inner cavity should be evaluated by subtracting two van der Waals radii of the hydrogen atom (2 × 1.2 Å) from this value, we estimated the effective inner diameter to be 4.6 Å. The calculated geometric characteristics of the Bu[6] molecule in the gas phase can be useful for further approximate size comparisons of potential guest molecules or ions with the bambus[6]uril host in the design of supramolecular systems.

**Figure 6:**
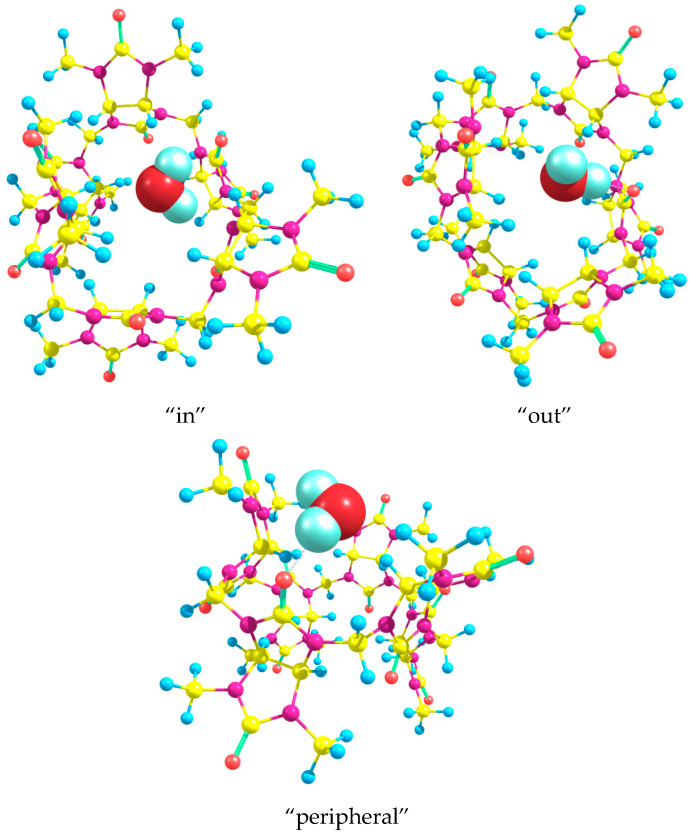
Structures of Bu[6]·H_2_O optimized using the DFT method. In the two upper panels, the water molecule forms a hydrogen bond with one of the equatorial oxygen atoms and is oriented inside (“in”) and outside (“out”) the cavity of bambus[6]uril. In the bottom panel, the water molecule forms a hydrogen bond with one of the peripheral oxygen atoms.

In the interaction with Bu[6], one water molecule preferably binds to the equatorial carbonyl oxygen atom, thus acting as a hydrogen bond (HB) donor. This “equatorial” binding mode allows the H_2_O molecule to further interact with the C-H bonds of neighboring glycoluril residues, thereby stabilizing the resulting Bu[6]·H_2_O monohydrate. Figure 6 depicts the optimized DFT structures of Bu[6] with the water molecule positioned in both “in” and “out” orientations within the Bu[6] cavity.

In order to compute the energy change for the hydration process, it is essential to have DFT results for both the Bu[6] and Bu[6]·H_2_O systems, as well as for the water molecule, which are obtained at the same level of theory. Given that water molecules exhibit strong association even in the gas phase [18], we employed a cluster model for water and performed calculations for the (H_2_O)_8_ and (H_2_O)_9_ clusters. Although water molecules easily form clusters of different sizes both in the liquid and gas phases, we chose the eight- and nine-molecule clusters, which have, respectively, cubic and modified cubic geometries [19] convenient for modeling due to their definite and non-flexible structures. Thus, the monohydrate formation process can be represented by the following scheme:

Bu[6] + (H_2_O)_9_ → Bu[6]·H_2_O + (H_2_O)_8_ (4)

Our DFT calculations show that the energy change ΔE for this process is determined to be −1.51 and −3.01 kcal/mol for the “in” and “out” structures of the product, respectively. The larger energy release obtained for the “out” monohydrate can be attributed to the formation of relatively strong additional C-H⋯OH_2_ hydrogen bonds. In the optimized “out” structure, the H⋯O distances between the water oxygen atom and the methine hydrogen atoms of neighboring glycoluril moieties are found to be 2.49 Å. Notably, the strongest hydrogen bond, which is formed between the water molecule and the equatorial carbonyl oxygen, measures 1.87 Å in length. We have also optimized the structures of monohydrate Bu[6]·H_2_O containing a water molecule bound in different orientations to one of the peripheral oxygen atoms. In the found low-energy “peripheral” monohydrate (Figure 6), the H⋯O hydrogen bond length equals 1.90 Å. The associated water molecule also acts as an HB donor to a nitrogen atom in one of the neighboring glycoluril fragments and as an HB acceptor to a CH hydrogen in another neighboring glycoluril moiety. The lengths of these weaker hydrogen bonds are 2.58 and 2.20 Å, respectively. However, the energy of the “peripheral” monohydrate is 4.06 kcal/mol higher in comparison with the “out” structure described above. Hence, the energy change ΔE for process (4) when the mono-hydration at the peripheral oxygen occurs can be easily calculated as +1.05 kcal/mol. This result is in agreement with our initial assumption that a water molecule preferably binds to the equatorial oxygen atom of Bu[6] than to the peripheral one.

**Figure 7:**
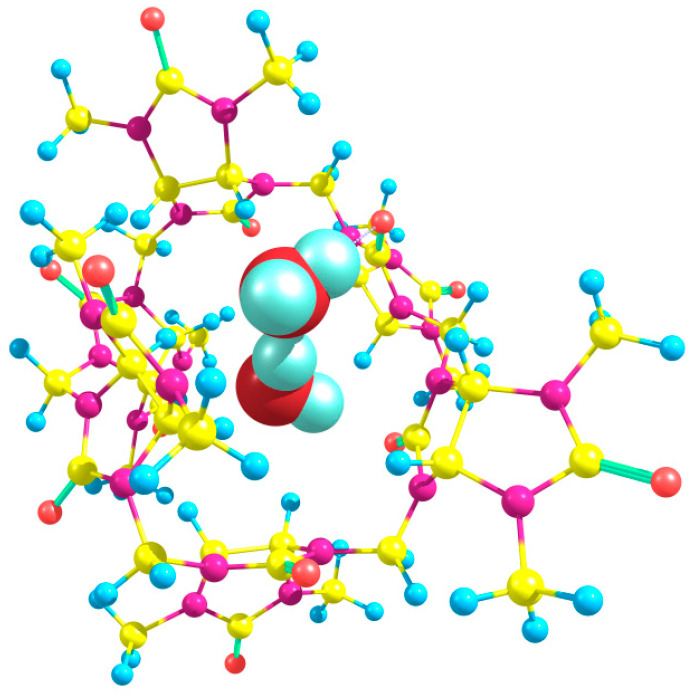
The structure of the most stable dihydrate Bu[6]·2H_2_O, corresponding to the out_in(*g*) arrangement, as determined using the DFT method.

Two water molecules upon binding to Bu[6] can participate in hydrogen bonding with the equatorial oxygen atoms in different relative positions (*syn*-, *gauche*-, and *trans*-) while adopting two distinct orientations (“in” and “out”). To explore the energetics, we performed calculations for dihydrates while considering various possible combinations of positions and orientations. Remarkably, the dihydrate with the lowest energy was found to possess an out_in(*g*) structure (Figure 7).

The dihydrate formation can be considered according to the following process (5) involving the water clusters:

Bu[6]·H_2_O + (H_2_O)_9_ → Bu[6]·2H_2_O + (H_2_O)_8_ (5)

The energies calculated using the DFT method for the starting compounds and products yield a value of ΔE = −4.65 kcal/mol for this process. Therefore, the overall energy change upon dihydrate formation starting from Bu[6] is −7.66 kcal/mol, indicating that the binding of two water molecules to Bu[6] is highly energetically favorable. In the out_in(*g*) dihydrate, the water molecules form hydrogen bonds with the equatorial carbonyl oxygen atoms (1.88 and 2.10 Å) as well as with each other (HB length of 1.89 Å). It is worth noting that hydrogen bonds between water molecules are also formed in other explored dihydrate structures, except for two out of the three structures with *trans*-oriented water molecules: out_in(*t*) and out_out(*t*). The calculated relative energies of the dihydrates span a considerable range, with the least stable out_in(*t*) dihydrate being 9.52 kcal/mol higher in energy compared to the out_in(*g*) structure.

**Figure 8:**
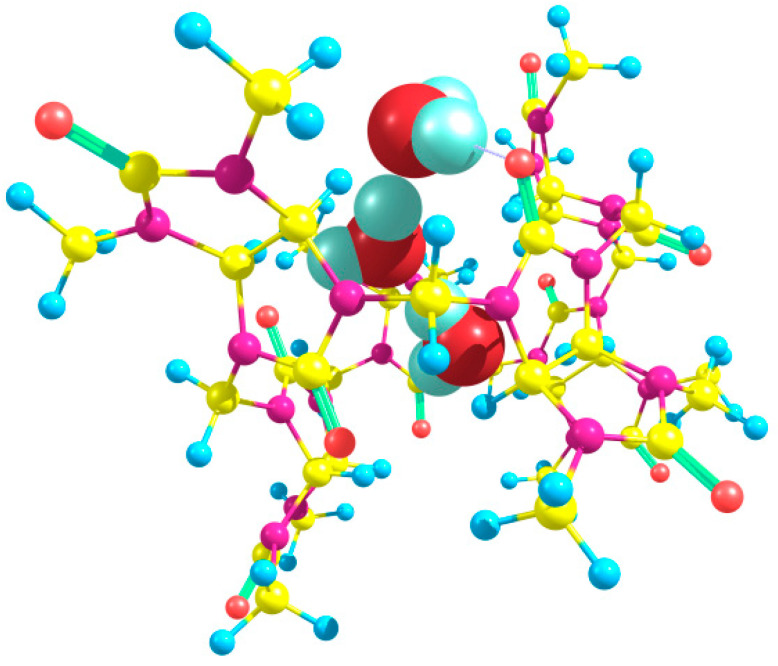
The structure of the most stable trihydrate Bu[6]·3H_2_O, corresponding to the out_out(*s*)_in(*t*) arrangement, as determined using the DFT method.

Trying to optimize the structures of trihydrate Bu[6]·3H_2_O with different relative positions and orientations of three water molecules bound to equatorial oxygen atoms, we obtained the energies of the corresponding aqua complexes varying within the interval of 4.54 kcal/mol. Among them, the out_out(*s*)_in(*t*) structure (Figure 8) had the lowest energy. It is noteworthy that the starting geometry before its optimization corresponded to the out_in(*g*)_in(*t*) arrangement and thus resembled a modification of the low-energy dihydrate out_in(*g*). However, during the optimization procedure, it was rearranged to out_out(*s*)_in(*t*).

The dihydrate to trihydrate conversion with the participation of water clusters can be described by process (6):

Bu[6]·2H_2_O + (H_2_O)_9_ → Bu[6]·3H_2_O + (H_2_O)_8_ (6)

The energy change ΔE calculated for reaction (6) is −0.90 kcal/mol. Thus, the energy release in this process is much lower than for the dihydrate formation via reaction (5). In the out_out(*s*)_in(*t*) trihydrate, two water molecules form strong hydrogen bonds with the equatorial oxygen atoms of Bu[6] with the H⋯O distances of 1.86 and 2.02 Å, whereas the third water molecule having the *trans*-“in” orientation forms a very weak HB with the *trans*-oxygen atom (H⋯O distance 3.15 Å) while it is hydrogen-bonded to the nearest nitrogen atom (H⋯N distance 2.43 Å). The three water guest molecules hosted by Bu[6] form hydrogen bonds with each other. The lengths of these bonds are 1.78 and 1.79 Å.

**Figure 9:**
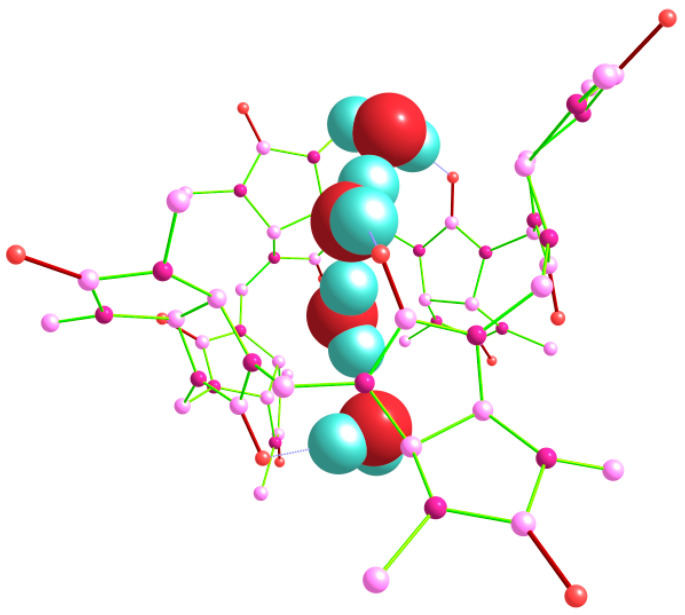
Structure of the tetrahydrate Bu[6]·4H_2_O, corresponding to the out_out(*s*)_w_out(*t*) arrangement, as determined using the DFT method. Hydrogen atoms in the Bu[6] molecule are not shown.

We have discovered that a linear chain of four hydrogen-bonded water molecules, (H_2_O)_4_, can also be encapsulated within the cavity of the bambus[6]uril host. The tetrahydrate Bu[6]·4H_2_O with the lowest energy was obtained based on the out_out(*s*)_in(*t*) trihydrate, and it can be classified as out_out(*s*)_w_out(*t*). In this arrangement, three water molecules form hydrogen bonds with the equatorial carbonyl oxygen atoms of the Bu[6] molecular container. However, one of the H_2_O molecules denoted as “w” is not hydrogen bonded to Bu[6], except a weak HB of the CH⋯OH_2_ type (H⋯O distance of 2.38 Å). The corresponding structure, optimized using the DFT method, is depicted in Figure 9. Our attempts to optimize Bu[6] tetrahydrates with other positions and orientations of the four H_2_O molecules led to higher-energy structures, the highest of them being the Bu[6] with the inclusion of a branched (H_2_O)_4_ guest cluster. This structure was 8.16 kcal/mol above the out_out(*s*)_w_out(*t*) tetrahydrate.

In the tetrahydrate structure with the out_out(*s*)_w_out(*t*) arrangement, the hydrogen bonds involving the carbonyl oxygen atoms exhibit lengths ranging from 1.81 to 1.95 Å. The hydrogen bonds between the water molecules themselves have lengths between 1.82 and 1.94 Å. The process (7) corresponds to the tetrahydrate formation from the trihydrate, involving the water clusters.

Bu[6]·3H_2_O + (H_2_O)_9_ → Bu[6]·4H_2_O + (H_2_O)_8_ (7)

According to the DFT results, the energy change associated with this process is ΔE = −3.20 kcal/mol. It is easy to calculate the value of ΔE = −4.10 kcal/mol for the tetrahydrate formation from dihydrate via the sequence of steps (6) and (7). This value indicates that the binding of two additional molecules following schemes (6) and (7) is less energetically favorable compared to the binding of the first two H_2_O molecules during the dihydrate formation (−4.10 vs. −7.66 kcal/mol). The obtained computational data agree with the results of the kinetic experiment presented above (Figure 3), indicating the rapid binding of two H_2_O molecules to the Bu[6] container, followed by the slower inclusion of two other water molecules.

**Figure 10:**
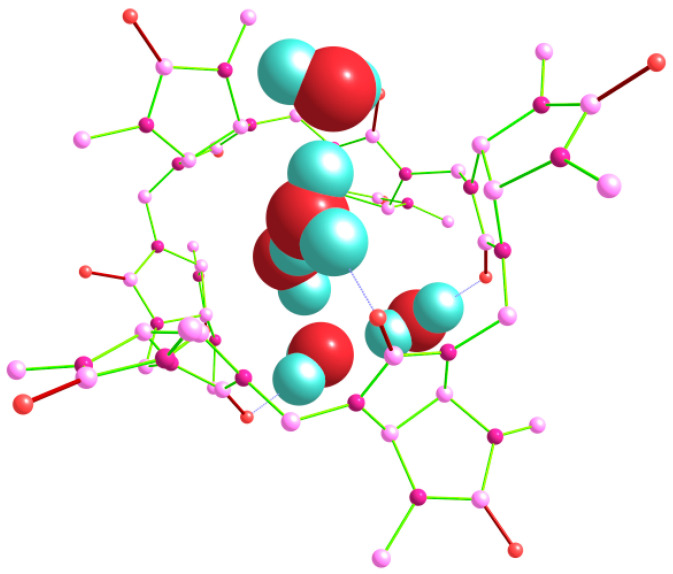
Structure of the pentahydrate Bu[6]·5H_2_O, corresponding to the out_out(*s*)_w_out(*t*)_in(*g*) arrangement, as determined using the DFT method. Hydrogen atoms in the Bu[6] molecule are not shown.

Finally, we tried to model several pentahydrate Bu[6]·5H_2_O structures, including those with branched and non-branched guest (H_2_O)_5_ clusters. The lowest energy after the DFT optimization corresponded to the structure classified as out_out(*s*)_w_out(*t*)_in(*g*) (Figure 10), which was based on the geometry of the lowest-energy tetrahydrate (see above).

The mono-hydration of the tetrahydrate giving the pentahydrate can be described by process (8):

Bu[6]·4H_2_O + (H_2_O)_9_ → Bu[6]·5H_2_O + (H_2_O)_8_ (8)

The value of ΔE = +1.23 kcal/mol was calculated for the pentahydrate formation according to Equation (8). Hence, further hydration of the tetrahydrate Bu[6]·4H_2_O becomes energetically unfavorable. This result can be explained by conformational peculiarities of Bu[6] necessary for the accomodation of five water molecules simultaneously. Indeed, for the inclusion of four or less H_2_O molecules, the Bu[6] container can be readily distorted to ensure efficient H⋯O, H⋯N, and CH⋯O hydrogen bond formation with the corresponding guest cluster. In the case of pentahydrate, the Bu[6] host is forced to become less distorted (see Figure 10), and it has lower possibilities for simultaneous hydrogen bonding with all of the captured water molecules.

It is more reasonable to analyze reaction thermodynamics rather than the ΔE values for hydration processes (4)–(8). For this purpose, we calculated the frequencies of normal vibrations by the DFT method for the optimized Bu[6] molecule, all the low-energy hydrates of Bu[6] (Figure 6, Figure 7, Figure 8, Figure 9 and Figure 10), and the water clusters (H_2_O)_8_ and (H_2_O)_9_. No imaginary frequencies were found in any case, which indicates the attainment of real energy minima for these structures upon the geometry optimizations. It should be noted that the contour of the DFT-simulated IR spectrum obtained with the Lorentzian broadening of peaks for Bu[6] in the gas phase contains bands of high and medium intensity at 1766, 1751, 1467, 1425, 1219, 1041, and 796 cm^−1^, which are in a satisfactory agreement with the experimental IR spectrum of a solid Bu[6] sample (see above). The thermodynamic characteristics (enthalpy ΔH°_298_ and Gibbs free energy ΔG°_298_) for the sequential reactions (4)–(8) were calculated based on the computed frequencies. These values, along with the energies ΔE, are shown in Table 1.

The ΔH°_298_ values of the sequential mono-, di-, tri-, and tetra-hydration indicate the exothermic nature of these processes, while the addition of the fifth water molecule is endothermic. The experimentally observed formation of the tetrahydrate from Bu[6] and four H_2_O molecules via processes (4)–(7) is highly exothermic according to the DFT data (ΔH°_298_ = −10.36 kcal/mol). The calculated Gibbs free energies ΔG°_298_ are less negative (or more positive) than the enthalpies ΔH°_298_ because of the unfavorable influence of entropy on all of the reactions (4)–(8). Nevertheless, the tetrahydrate formation from anhydrous Bu[6] through the sequence of these reactions has a totally negative ΔG°_298_ sum of −7.42 kcal/mol, in spite of the positive free energy change in stage (6). Importantly, for the first two stages, (4) and (5), of the dihydrate formation, the sum of Gibbs energies equals −5.81 kcal/mol, while for the following two stages, (6) and (7), the corresponding ΔG°_298_ sum is −1.61 kcal/mol. This result is in good agreement with the observed rapid formation of dihydrate Bu[6]·2H_2_O followed by its slower conversion to tetrahydrate Bu[6]·4H_2_O (Section 2.2), although we did not estimate energy barriers for the sequential hydration reactions.

Thus, the DFT method can be regarded as a powerful tool for the description of geometric and thermodynamic properties of supramolecular systems based on bambus[6]uril.

## 3. Materials and Methods

The infrared (IR) spectrum was recorded using a Nicolet 6700 Fourier Transform Infrared (FT-IR) spectrometer (Thermo Electron Corporation, Waltham, MA, USA) equipped with a diamond crystal and an attachment for attenuated total reflectance (ATR). The spectral resolution was set at 4 cm^−1^, with 64 scans collected over the range of 400–4000 cm^−1^.

For nuclear magnetic resonance (NMR) analysis, a Bruker AVANCE 400 IIIHD NMR spectrometer (Bruker, Billerica, MA, USA) was utilized. One-dimensional spectra were acquired for both proton (^1^H) and carbon (^13^C) nuclei at frequencies of 400.17 MHz and 100.63 MHz, respectively. A DMSO-d_6_/CDCl_3_ mixture in a 1:1 ratio was used as the solvent.

### DFT Calculations

The geometry optimizations of Bu[6], its hydrates, and water clusters were performed by the composite DFT method B97-3c [17] using the ORCA 5.0.2 software [20] on a server (16 × 2.4 GHz CPU, 16 Gb RAM) operating under Ubuntu 20.04. No implicit solvation model or symmetry constraints were applied. The Bu[6] molecule, water clusters (H_2_O)_8_, (H_2_O)_9_, and low-energy structure of each type of hydrate (mono-, di-, tri-, tetra-, and penta-hydrated bambus[6]uril) were subjected to further normal vibration analysis and thermochemistry calculations using the DFT approximation mentioned above. Enthalpies and Gibbs free energies were calculated using the Quasi-RRHO approach [21], which provides the most correct account for low-frequency normal vibrations, which is especially important in calculations of supramolecular systems. The visualization of the results and the simulation of IR bands were performed with the use of Chemcraft 1.8 software [22].

## 4. Conclusions

In this study, we investigated for the first time the absorption/desorption capacity of bambus[6]uril towards water using the gravimetric method with the use of McBain–Bakr quartz spring balances. It was found that the absorption capacity of Bu[6] was 4 moles of water per mole of bambus[6]uril, and the time to reach saturation at 25°C was 20 min, while the time for complete removal of water was 100 min. The pseudo-first-order model was employed to calculate the absorption and desorption rate constants for these processes, resulting in values of *k_abs_* = 0.166 ± 0.008 min^−1^ and *k_des_* = 0.0221 ± 0.0013 min^−1^, respectively.

The DFT calculations revealed that the binding of water molecules to bambus[6]uril occurs through the formation of hydrogen bonds between the equatorial oxygen atoms of Bu[6] and the hydrogen atoms of water molecules. The formation of the dihydrate was found to be thermodynamically more favorable compared to binding two additional water molecules to form the tetrahydrate. These computational results are in agreement with the experimental observations and indicate that the DFT method can be a useful tool for predicting and interpreting experimental data regarding the formation of inclusion complexes with the bambus[6]uril molecular container.

Our studies suggest the structures of bambus[6]uril aqua complexes. The presence of strongly bound water molecules in the inner cavity of bambus[6]uril can affect the formation and stabilities of supramolecular host–guest compounds.

## Figures and Tables

**Table 1:** Values of ΔE, ΔH°_298_, and ΔG°_298_ (in kcal/mol) for the hydration processes (4)*–*(8) calculated using the DFT method.

Reaction	Product	ΔE	ΔH°_298_	ΔG°_298_
(4)	Bu[6]·H_2_O	−3.01	−2.96	−2.58
(5)	Bu[6]·2H_2_O	−4.65	−4.23	−3.23
(6)	Bu[6]·3H_2_O	−0.90	−0.39	+0.67
(7)	Bu[6]·4H_2_O	−3.20	−2.78	−2.28
(8)	Bu[6]·5H_2_O	+1.23	+1.51	+2.28

## Data Availability

The data presented in this study are available in this article.

## Supplementary Materials

The following supporting information can be downloaded at: https://www.mdpi.com/article/10.3390/molecules28237680/s1, Figures S1 and S2 with the linear plots corresponding to equations (2) and (3); ZIP archive with ORCA output files containing the DFT-optimized 3D structures of Bu[6], its low-energy hydrates, and water clusters. Figure S1. Plot of the experimental data for the absorption kinetics of water vapor by Bu[6]. Axis x: time, minutes. Axis y: ln (α_*max*_ − α); Figure S2. Plot of the experimental data for the desorption kinetics of water vapor from the water-saturated Bu[6]. Axis x: time, minutes. Axis y: ln α.
