# Supplementary material for: Absorption of Water Vapor by Bambus[6]uril and a Density Functional Theory Study of Its Aqua Complexes
Source: Molecules. 2023 Nov 21;28(23):7680. doi: 10.3390/molecules28237680 (PMC10708022; doi:10.3390/molecules28237680)
Supplement: Supplementary file 1 [file molecules-28-07680-s001.zip › Figures_S1_and_S2.pdf]

Absorption of Water Vapor by Bambus[6]uril and a Density Functional Theory Study of Its Aqua Complexes

Pana Turebayeva [1,]*, Alexey N. Guslyakov [2], Svetlana A. Novikova [3], Andrei I. Khlebnikov [3], Ekaterina A. Befus [2], Evgeniy P. Meshcheryakov [2], Abdigali A. Bakibaev [2], Lyazat Kusepova [1], Nazira Kassenova [4] , Sarzhan Sharipova [5] and Rakhmetulla Yerkassov [1]

[1] Department of Chemistry, L.N. Gumilyov Eurasian National University, 010008 Astana, Kazakhstan (kusepova71@mail.ru)

[2] Faculty of Chemistry, National Research Tomsk State University, 634050 Tomsk, Russia (A.N.G. guslyakov.aleksej@bk.ru, E.A.B.:ekaterina.befus@mail.ru, E.P.M.: meevgeni@mail.ru)

[3] Kizhner Research Center, Tomsk Polytechnic University, 634050 Tomsk, Russia (san22@tpu.ru)

[4] Department of Chemistry and Biotechnology, Sh. Ualikhanov Kokshetau University, 020000 Kokshetau, Kazakhstan (nazira09_83@mail.ru)

[5] Department of Pharmaceutical and Toxicological Chemistry, Pharmacognosy and Botany, Asfendiyarov Kazakh national medical university, 050000 Almaty, Kazakhstan

* Correspondence: pana90@mail.ru; Tel.: +7-707-3488487

Supplementary information

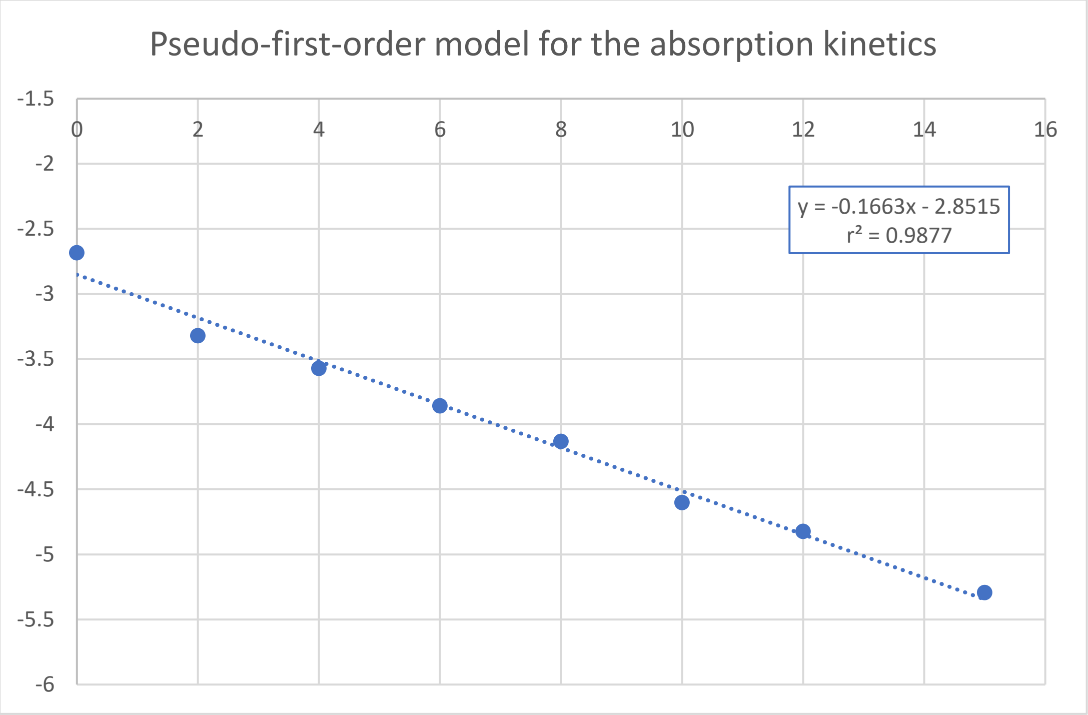

Figure S1. Plot of the experimental data for the absorption kinetics of water vapor by Bu[6]. Axis **x**: time, minutes. Axis **y**: $\ln(\alpha_{max} - \alpha)$.

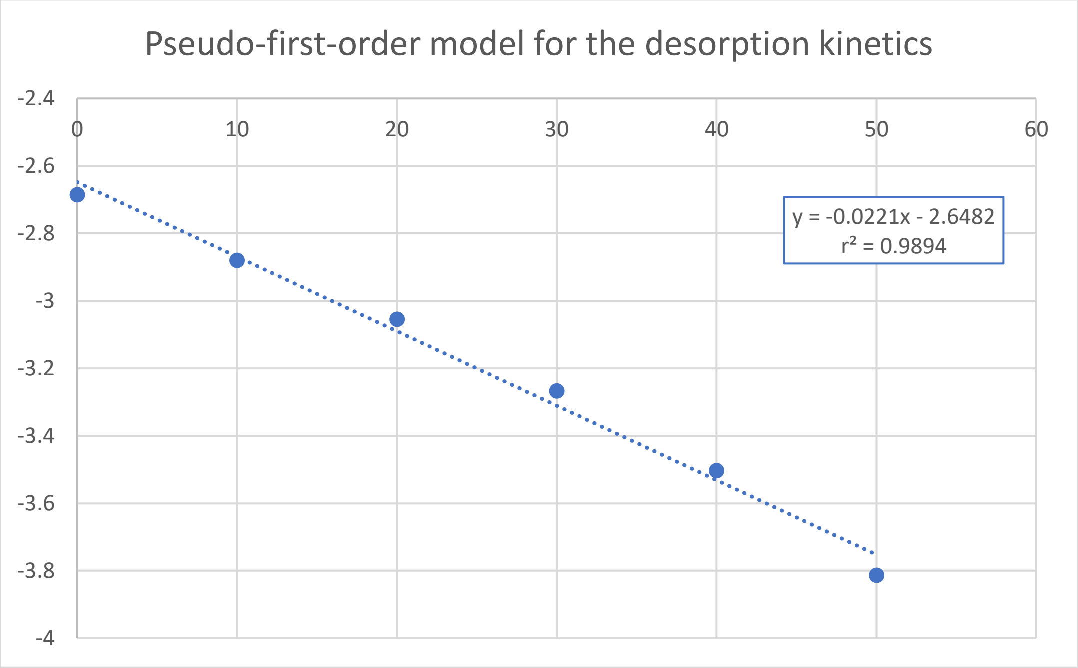

Figure S2. Plot of the experimental data for the desorption kinetics of water vapor from the water-saturated Bu[6]. Axis **x**: time, minutes. Axis **y**: ln α.
